# Effects of UMP, Choline, and Fish Oil on Synaptic Integrity and Motor Coordination in an Alzheimer’s Disease Mouse Model

**DOI:** 10.3390/ijms27083342

**Published:** 2026-04-08

**Authors:** Elif Nedret Keskinoz, Ghazal Footohi, Musa Celik, Dilan Acar, Gokcen Ozgun, Merve Acikel Elmas, İlayda Yavuz, Ece Ada, Efe Sari, Beril Ay, Mehmet Can Unal, İsmail Hakki Ulus, Serap Arbak, Guldal Suyen, Devrim Oz-Arslan

**Affiliations:** 1 Department of Anatomy, School of Medicine, Acibadem Mehmet Ali Aydinlar University, 34752 Istanbul, Turkey; elif.keskinoz@acibadem.edu.tr; 2 Department of Neuroscience, Graduate School of Health Sciences, Acibadem Mehmet Ali Aydinlar University, 34752 Istanbul, Turkey; ghazalfootohi22@gmail.com (G.F.); guldal.suyen@acibadem.edu.tr (G.S.); 3 Department of Anatomy, Graduate School of Health Sciences, Acibadem Mehmet Ali Aydinlar University, 34752 Istanbul, Turkey; 4 Department of Biophysics, Graduate School of Health Sciences, Acibadem Mehmet Ali Aydinlar University, 34752 Istanbul, Turkey; mmusacelik7@gmail.com; 5 Department of Physiology, Graduate School of Health Sciences, Acibadem Mehmet Ali Aydinlar University, 34752 Istanbul, Turkey; acaar.dilan@gmail.com; 6 Department of Medical Biotechnology, Graduate School of Health Sciences, Acibadem Mehmet Ali Aydinlar University, 34752 Istanbul, Turkey; gokcen.ozgun@acibadem.edu.tr; 7 Department of Histology and Embryology, School of Medicine, Acibadem Mehmet Ali Aydinlar University, 34752 Istanbul, Turkey; merve.elmas@marmara.edu.tr; 8 Department of Histology and Embryology, Marmara University School of Medicine, 34854 Istanbul, Turkey; serap.arbak@acibadem.edu.tr; 9 School of Medicine, Acibadem Mehmet Ali Aydinlar University, 34752 Istanbul, Turkey; ilaydaesmayavuzz@gmail.com (İ.Y.); eceada99@gmail.com (E.A.); efessarri@gmail.com (E.S.); berilay.md@gmail.com (B.A.); 10 Department of Molecular Biology and Genetics, Faculty of Engineering and Natural Sciences, Acibadem Mehmet Ali Aydinlar University, 34752 Istanbul, Turkey; mehmet.unal@live.acibadem.edu.tr; 11 Department of Medical Pharmacology, School of Medicine, Acibadem Mehmet Ali Aydinlar University, 34752 Istanbul, Turkey; ismail.ulus@okan.edu.tr; 12 Department of Medical Pharmacology, Faculty of Medicine, Istanbul Okan University, 34959 Istanbul, Turkey; 13 Department of Physiology, School of Medicine, Acibadem Mehmet Ali Aydinlar University, 34752 Istanbul, Turkey; 14 Department of Biophysics, School of Medicine, Acibadem Mehmet Ali Aydinlar University, 34752 Istanbul, Turkey

**Keywords:** uridine monophosphate, choline, docosahexaenoic acid, fish oil, synaptic integrity, 5XFAD mouse model, behavioral performance

## Abstract

Alzheimer’s disease (AD) is an age-related neurodegenerative disorder characterized by progressive synaptic dysfunction, axonal pathology, and cognitive decline, with the hippocampal circuits showing particular vulnerability during disease progression. However, early-life nutritional interventions may influence long-term synaptic resilience. In this study, we investigated the long-term effects of prenatal and lactational supplementation with choline, UMP, and fish oil in the 5XFAD mouse model. To this end, hippocampal synaptic and axonal pathology was assessed at 3, 6, and 9 months using Western blotting and immunofluorescence to measure synaptophysin, PSD-95, and neurofilament medium chain (NF-M), alongside a multidimensional behavioral battery that evaluated cognitive, affective, motor, and sensory outcomes. Results showed that early-life supplementation did not significantly improve the learning performance decline, increase nociception, or reverse changes in anxiety behavior in transgenic mice. However, it attenuated synaptic decline in transgenic animals by partially preserving synaptophysin and PSD-95 levels and reducing NF-M elevations. These molecular effects were accompanied by selective behavioral modulation, including preserved learning dynamics, altered anxiety-like behavior, and delayed nociceptive hypersensitivity, while late-stage motor impairments remained largely unaffected. Overall, prenatal and lactational supplementation produced modest, age-dependent effects on synaptic markers and partially prevented neurodegenerative progression in the 5XFAD model.

## 1. Introduction

Alzheimer’s disease (AD) is a neurodegenerative disorder characterized pathologically by amyloid-β (Aβ) accumulation, neurofibrillary tangles, synaptic alterations, axonal degeneration, and neuronal loss, leading clinically to cognitive decline. The hippocampal circuits, which form the basis of learning and memory, are known to be particularly vulnerable to these pathological processes [1,2,3,4,5]. Accumulated studies have reported impairments in dendritic morphology and synaptic loss in AD, characterized by reduced synaptic protein markers and density, which are closely associated with cognitive deterioration [5,6,7,8,9,10,11].

Nutritional factors have been shown to influence brain structure and function, and several nutrients are known to be essential for normal neuronal development and synaptic maintenance [12,13]. The prenatal and early postnatal periods are critical phases of brain development, during which nutritional availability contributes to neuronal differentiation, synapse formation, and long-term brain structure [14]. Among these nutrients, choline, uridine, and fish oil are nutrients that play complementary roles in membrane synthesis and synaptogenesis. Choline is required for normal fetal brain development [15]; uridine contributes to neurodevelopmental processes, including membrane synthesis and synapse formation [13]; and docosahexaenoic acid (DHA) is associated with synaptic density and cognitive performance in offspring [16]. Accordingly, choline, uridine monophosphate (UMP), and fish oil have been studied both individually and in combination, and supplementation with these nutrients has been demonstrated to modulate synaptic protein expression and cognitive performance in both experimental and clinical studies [13,17,18,19].

Given the critical role of synapses in learning and memory [20], preserving synaptic integrity or supporting synaptogenesis during the lactation period may enhance neurotransmission, improve synaptic function, and promote cognition in offspring [12]. Despite evidence that choline, UMP, and fish oil support neurodevelopment and synaptic function, it remains unclear whether the changes in synaptic integrity and behavioral outcomes are maintained long-term when these supplements are provided during the prenatal or lactation period. To address this knowledge gap, we investigated the long-term effects of combined dietary supplementation with choline, UMP, and fish oil administered exclusively during the prenatal and lactational periods in the 5XFAD mouse model of AD and control animals. Cognitive and behavioral performance was first assessed using a comprehensive battery of tests to determine whether early-life nutritional supplementation was associated with sustained alterations in behavioral phenotypes.

Subsequently, hippocampal levels of key synaptic and axonal markers (including PSD-95, synaptophysin (SYP), and neurofilament medium chain (NF-M)) were examined in a region-specific manner within the CA1 and CA3 subfields at 3, 6, and 9 months of age. To our knowledge, this study provides one of the first integrated evaluations examining how early-life nutritional supplementation with choline, UMP, and fish oil influences behavioral outcomes together with presynaptic, postsynaptic, and axonal markers in the 5xFAD model. By combining behavioral phenotyping with region-specific analyses of synaptic and axonal markers in hippocampal CA1 and CA3 subfields, this work extends previous studies that have typically examined these parameters separately. Overall, these findings extend existing evidence by demonstrating that nutritional exposure confined to early developmental stages can shape long-term molecular integrity and functional trajectories later in life.

## 2. Results

### 2.1. Cognitive and Behavioral Assessment of 5XFAD and Control Mice Following Prenatal and Lactational Nutritional Supplementation

Cognitive and behavioral testing provides a means to relate molecular and synaptic changes to disease-relevant phenotypes in animal models of AD. The 5xFAD mouse exhibits alterations across multiple cognitive and behavioral domains, including learning and memory, motor coordination, anxiety-related behavior, and sensory processing [21]. Behavioral outcomes were evaluated across four experimental groups: control diet non-transgenic (CNTR), control diet transgenic (CTR), experimental diet non-transgenic (ENTR), and experimental diet transgenic (ETR) mice.

#### 2.1.1. Locomotor Activity and Exploratory Behavior (Open Field Test)

**Figure 1:**
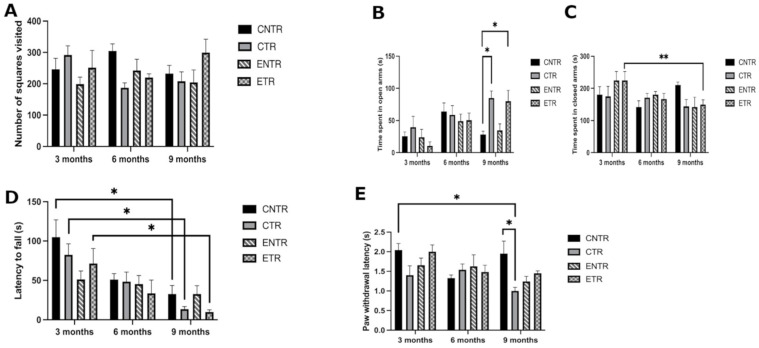
Behavioral assessments in Alzheimer’s disease. Effects of prenatal and lactational nutritional supplementation on locomotor activity, anxiety-like behavior, motor coordination, and nociceptive response in 5XFAD mice. (**A**) Open field test: locomotor activity was expressed as the number of squares visited. (**B**,**C**) Elevated plus maze test: time spent in open arms (**B**) and closed arms (**C**) was measured. (**D**) Rotarod test: motor coordination was expressed as the falling latency (seconds) from an accelerating rotarod (4–40 rpm over 5 min). (**E**) Paw withdrawal test: paw withdrawal latency was measured (seconds). Data is presented as the mean ± standard error of the mean (SEM) (*n* = 5–7 animals per group). Statistical significance is indicated as * *p* < 0.05 and ** *p* < 0.01.

Spontaneous locomotor activity was assessed using the open field test by measuring the total number of squares visited at 3, 6, and 9 months of age in the CNTR, CTR, ENTR, and ETR groups. No statistically significant differences were observed among the groups at any time point (Figure 1A). These findings indicate that locomotor activity remained preserved across groups and suggest that the differences observed in other behavioral paradigms were unlikely to be attributable to major changes in basal locomotion.

#### 2.1.2. Anxiety-like Behavior (Elevated Plus Maze)

Anxiety-like behavior was assessed using an elevated plus maze by measuring the time spent in the open and closed arms at 3, 6, and 9 months of age. In the 3- and 6-month assessments, the duration of time spent in the open arms was not statistically different between the four groups. In contrast, at the 9-month time point, both the CTR and the ETR groups exhibited significantly increased open-arm exploration compared to the CNTR group (*p* < 0.05) (Figure 1B). This pattern is consistent with reduced anxiety-like avoidance or altered exploratory behavior in transgenic animals at the later disease stage.

No significant differences were observed among the groups in the time spent in the closed arms at any time point. However, within-group analysis revealed that the ETR group spent significantly less time in the closed arms at 9 months compared to 3 months (*p* < 0.01) (Figure 1C).

#### 2.1.3. Motor Coordination and Balance (Rotarod Test)

Motor coordination and balance were assessed using the rotarod test to reveal any age-dependent changes across the groups. There was no difference among the groups in terms of falling latency in all assessments. However, statistical comparisons across ages revealed a significant decline in motor performance in the CNTR, CTR, and ETR groups at 9 months compared to 3 months (*p* < 0.05), indicating an age-related deterioration in motor coordination (Figure 1D). Although the graphical distribution suggested a tendency toward reduced performance in transgenic animals at 9 months of age, these differences did not reach statistical significance.

#### 2.1.4. Pain Sensitivity/Nociceptive Response (Paw Withdrawal Test)

Withdrawal latency in response to thermal stimulation was measured to assess the thermal sensitivity of the mice in the four groups. However, at 9 months of age, the average paw withdrawal latency was significantly decreased in the CTR group compared to the CNTR group (*p* < 0.05), indicating an increased sensitivity in the transgenic animals at the later disease stage to thermal stimulus. There was no difference in thermal sensitivity among groups at the 3- or 6-month time points (Figure 1E).

#### 2.1.5. Spatial Learning and Memory (Morris Water Maze)

**Figure 2:**
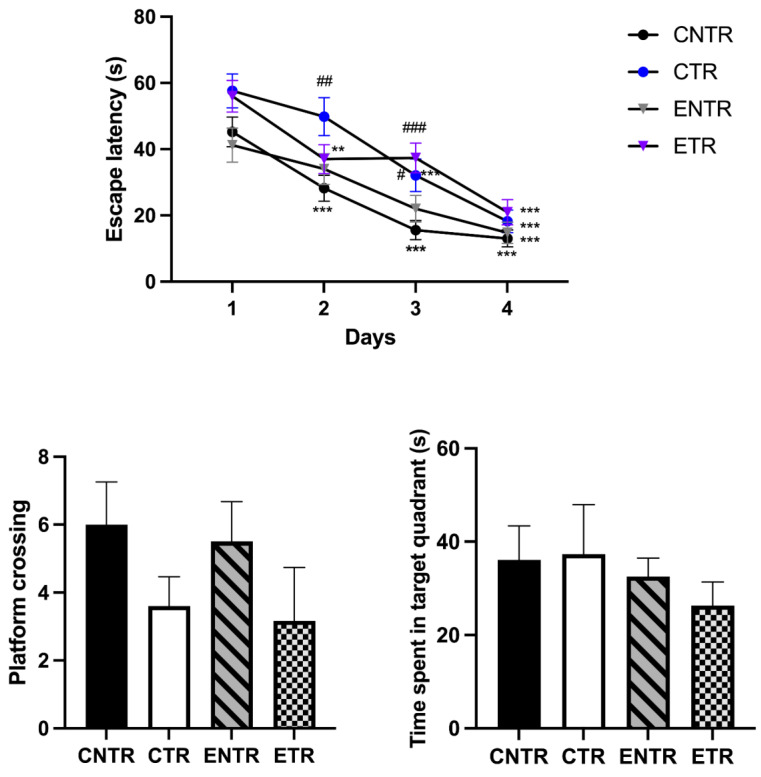
Learning and memory performance assessment in Alzheimer’s disease. Effects of prenatal and lactational dietary supplementation on spatial learning and memory in 9-month-old mice assessed using the Morris water maze (MWM) test. (**A**) Escape latency during the acquisition phase, (**B**) number of platform crossings, and (**C**) time spent in the target quadrant during the probe trial. Data are presented as the mean ± SD, with each point representing an individual animal. Escape latency across training days was analyzed using repeated-measures ANOVA, with the training day used as the within-subject factor, followed by Bonferroni correction. Tukey’s post hoc test was applied for pairwise comparisons. Data are presented as the mean ± SEM (*n* = 5–7 animals per group). ** *p* < 0.01, *** *p* < 0.001 vs. day 1. # *p* < 0.05, ## *p* < 0.01, ### *p* < 0.001 vs. CNTR group. CNTR, control group (black circles); CTR transgenic group (blue circles); ENTR, control group receiving dietary supplementation (gray downward triangles); ETR, transgenic group receiving dietary supplementation (purple downward triangles).

Spatial learning and memory were assessed using the Morris water maze (MWM) test at 9 months of age. Analysis of daily escape latency in all groups (CNTR, CTR, ENTR, and ETR) revealed a significant decrease in the mean latency to locate the platform on the fourth training day compared with the first day (*p* < 0.001). This result indicated that the mice in all groups learned the task. However, the learning performance on the second and third days varied considerably. In the CNTR group, the average escape latency was significantly lower on the second and third days compared to the initial training day (*p* < 0.001). In the CTR group, a significant reduction in escape latency occurred on the third day (*p* < 0.001), indicating slower acquisition compared to the CNTR group. The ETR group showed a better performance on the second day (*p* < 0.01), which was not observed on the third day. Finally, in the ENTR group, the escape latencies for the second and third days were not significantly different from those of the first day (Figure 2A).

Daily between-group comparisons of escape latency further supported altered acquisition dynamics in transgenic animals. The average escape latency in the CTR group was significantly longer than that of the CNTR group on the second day (*p* < 0.01), while both the CTR (*p* < 0.05) and ETR (*p* < 0.001) groups exhibited significantly longer escape latencies than the CNTR group on the third day. Together, these findings indicate that transgenic mice, particularly the non-supplemented group, displayed slower learning during the acquisition phase.

In the probe trial, no significant differences were observed among groups regarding the number of platform crossings or the time spent in the target quadrant (Figure 2B,C). Although acquisition dynamics differed across groups, probe trial performance remained relatively preserved, suggesting that delayed learning did not translate into impairment in spatial reference memory at this stage.

### 2.2. Membrane Lipid Precursors Modulate Hippocampal Synaptic Organization in the 5XFAD Mouse Model

To further assess the effects of nutrient supplementation on region-specific alterations in synaptogenesis and synaptic network organization, immunoreactivity of SYP, PSD-95, and NF-M was assessed in the CA1 and CA3 regions of the hippocampus using immunofluorescence and Western blot analyses. Synaptophysin is a presynaptic vesicle protein widely used as an indicator of synaptic terminal density and neurotransmission capacity [22]. PSD-95, a key postsynaptic scaffolding protein that stabilizes glutamatergic synapses, is essential for maintaining synaptic integrity [23]. In addition, NF-M, a core component of the axonal cytoskeleton, serves as a marker of axonal integrity and neurodegeneration in AD, reflecting axonal health and conduction properties and contributing to protection against oxidative damage [24].

### 2.3. Early Life Supplementation Partially Preserves Presynaptic Protein Levels (Synaptophysin)

**Figure 3:**
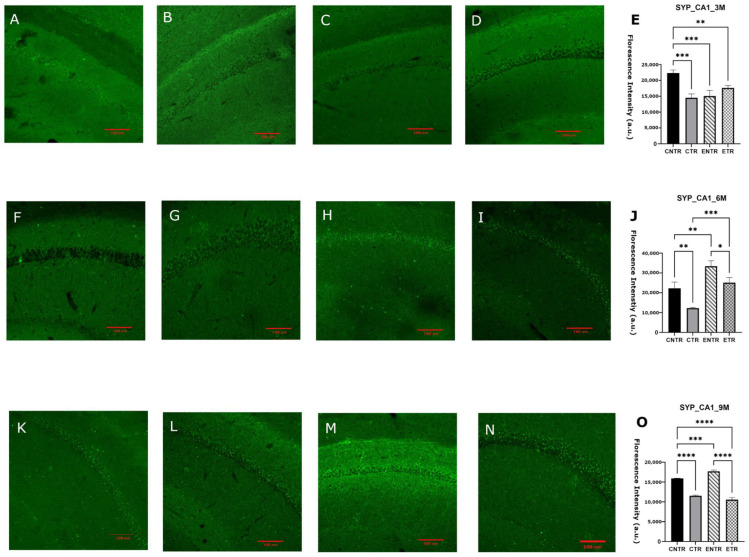
Analysis of synaptophysin (SYP) expression in the hippocampal CA1 region at 3, 6, and 9 months using immunofluorescence. Representative images for CNTR, CTR, ENTR, and ETR groups are shown at 3 months (**A**–**D**), 6 months (**F**–**I**), and 9 months (**K**–**N**). Corresponding quantification of SYP fluorescence intensity density is presented in (**E**), (**J**), and (**O**), respectively. Data are expressed as mean ± SEM (*n* = 3 animals per group). Statistical significance was determined using one-way ANOVA followed by Tukey’s post hoc test. * *p* < 0.05, ** *p* < 0.01, *** *p* < 0.001, **** *p* < 0.0001. Images were acquired at 20× magnification; scale bar = 100 μm.

**Figure 4:**
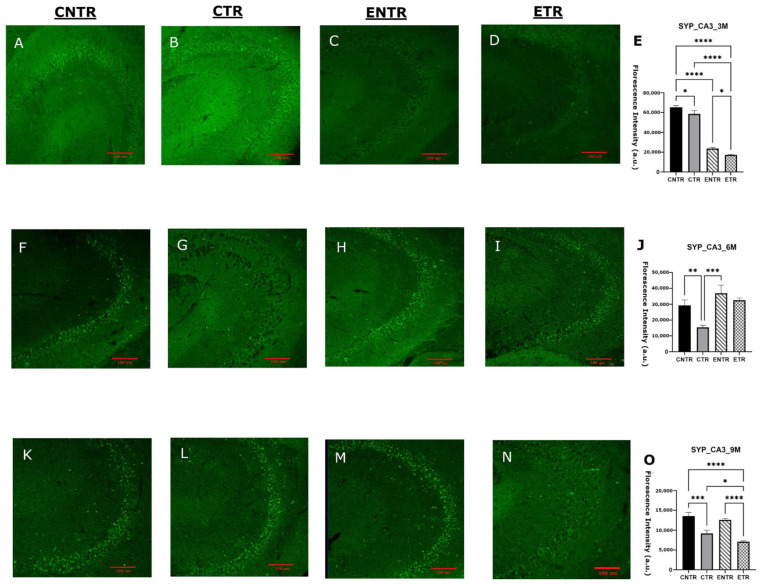
Analysis of synaptophysin (SYP) expression in the hippocampal CA3 region at 3, 6, and 9 months using immunofluorescence. Representative SYP immunofluorescence images are shown for 3 months (**A**–**D**), 6 months (**F**–**I**), and 9 months (**K**–**N**), with corresponding quantitative analyses presented in (**E**), (**J**), and (**O**), respectively. Data are presented as the mean ± SEM (*n* = 3 animals per group). Statistical analysis was performed using one-way ANOVA followed by Tukey’s post hoc test. * *p* < 0.05, ** *p* < 0.01, *** *p* < 0.001, **** *p* < 0.0001. Images were acquired at 20× magnification; scale bar = 100 μm.

**Figure 5:**
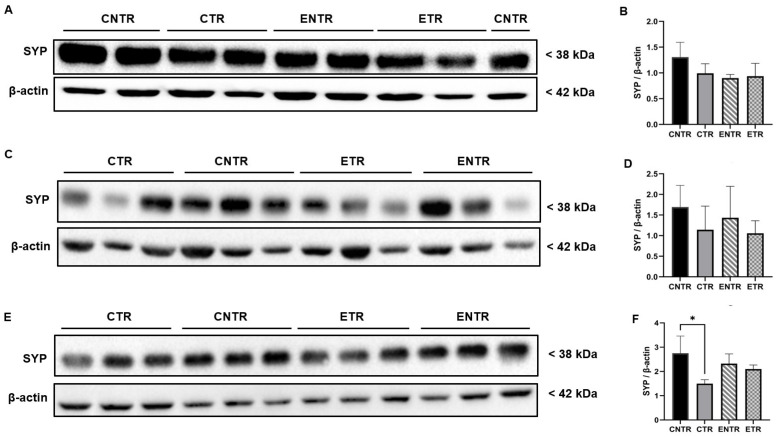
Analysis of synaptophysin (SYP) expression in hippocampal homogenates at 3, 6, and 9 months using Western blot. Representative Western blot images of SYP expression (3 months, (**A**); 6 months, (**C**); 9 months, (**E**)). Quantification of SYP band intensities normalized to β-actin for the corresponding age groups (**B**,**D**,**F**), respectively. Data are presented as the mean ± SEM (*n* = 4 animals per group). Statistical analysis was performed using one-way ANOVA followed by Tukey’s post hoc test. * *p* < 0.05.

Immunofluorescence analysis of the hippocampal CA1 and CA3 regions revealed significant group-dependent differences in SYP mean fluorescence intensity. In both regions, CNTR animals exhibited markedly higher SYP levels than the other groups at 3 months of age. At 6 months, SYP intensity was downregulated in the CTR group, resulting in significantly lower levels compared to the CNTR group, whereas ETR animals displayed relatively preserved SYP intensity. By 9 months, the group differences became more pronounced: SYP levels were highest in the control and experimental groups, while both transgenic groups displayed a reduction in SYP (Figure 3O and Figure 4O). To determine whether these regional differences were reflected at the whole-tissue level, SYP protein levels were subsequently quantified in total hippocampal homogenates. In line with the immunofluorescence findings, no statistically significant differences in SYP expression were detected at 3 or 6 months of age, although minor variations were observed by Western blot analysis. By 9 months of age, however, CTR animals displayed a significant downregulation in SYP expression compared to CNTR animals, supporting the reduction in synaptic marker density observed by immunofluorescence in transgenic mice (Figure 5).

### 2.4. Postsynaptic Marker PSD-95: An Age-Dependent Decline That Can Be Attenuated by Supplementation

**Figure 6:**
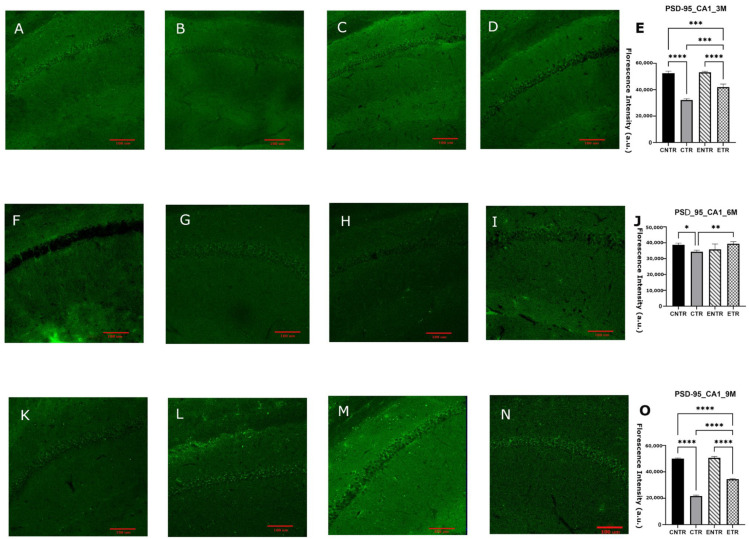
Immunofluorescence images and quantitative analysis of PSD-95 expression in the hippocampal CA1 region at 3, 6, and 9 months of age. Representative images for CNTR, CTR, ENTR, and ETR groups are shown at 3 months (**A**–**D**), 6 months (**F**–**I**), and 9 months (**K**–**N**). Corresponding quantification of PSD-95 fluorescence intensity density is presented in (**E**), (**J**), and (**O**), respectively. Data are expressed as mean ± SEM (*n* = 3 animals per group). Statistical significance was determined by one-way ANOVA with Tukey’s post hoc test. * *p* < 0.05, ** *p* < 0.01, *** *p* < 0.001, **** *p* < 0.0001. Images were acquired at 20× magnification; scale bar = 100 μm.

**Figure 7:**
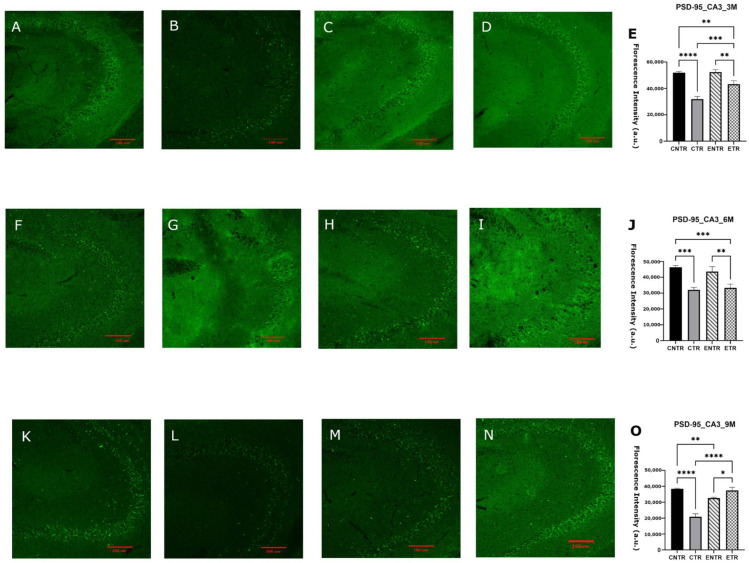
Immunofluorescence images and quantitative analysis of PSD-95 expression in the hippocampal CA3 region at 3, 6, and 9 months of age. Representative PSD-95 immunofluorescence images are shown for 3 months (**A**–**D**), 6 months (**F**–**I**), and 9 months (**K**–**N**), with corresponding quantitative analyses presented in (**E**), (**J**), and (**O**), respectively. Data are presented as the mean ± SEM (*n* = 3 animals per group). Statistical analysis was performed using one-way ANOVA followed by Tukey’s post hoc test. * *p* < 0.05, ** *p* < 0.01, *** *p* < 0.001, **** *p* < 0.0001. Images were acquired at 20× magnification; scale bar = 100 μm.

**Figure 8:**
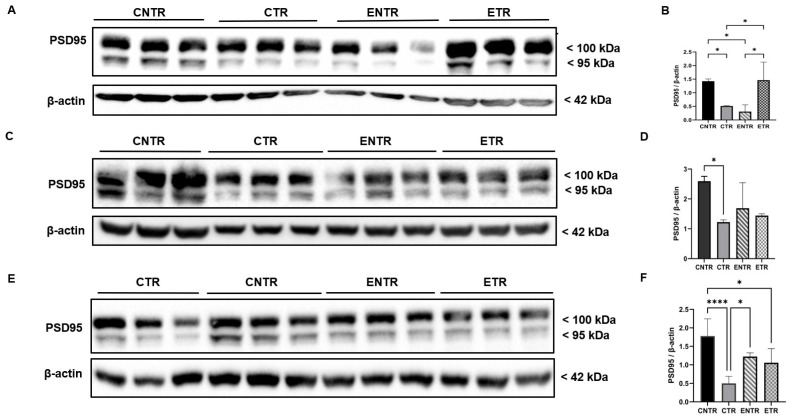
Western blot analysis of PSD-95 expression in hippocampal homogenates at 3, 6, and 9 months of age. Representative Western blot images of PSD-95 expression (3 months, (**A**); 6 months, (**C**); 9 months, (**E**)). Quantification of PSD-95 band intensities normalized to β-actin for the corresponding age groups (**B**,**D**,**F**), respectively. Data are presented as the mean ± SEM (*n* = 4 animals per group). Statistical analysis was performed using one-way ANOVA followed by Tukey’s post hoc test. * *p* < 0.05, **** *p* < 0.0001.

Immunofluorescence analysis of the hippocampal CA1 and CA3 regions revealed significant group-dependent differences in mean fluorescence intensity of PSD-95. In all age groups, CNTR animals exhibited higher PSD-95 intensity compared to mice in the CTR and ETR groups; ETR animals also showed higher levels than CTR mice. The difference between transgenic and non-transgenic groups was markedly elevated at 9 months of age (Figure 6O), whereas ETR animals displayed statistically significant levels of PSD-95 compared to mice in the CTR group at 6 and 9 months of age in the CA1 and 6-month CA3 regions (Figure 7O). Western blot analysis of PSD-95 expression in hippocampal homogenates revealed that CNTR animals exhibited the highest levels of PSD-95 among all groups. By 9 months of age, CNTR animals exhibited markedly higher PSD-95 levels relative to CTR and modestly higher expression compared to ETR (Figure 8).

### 2.5. Axonal Pathology Reflected by NF-M Elevation Is Downregulated by Supplementation

**Figure 9:**
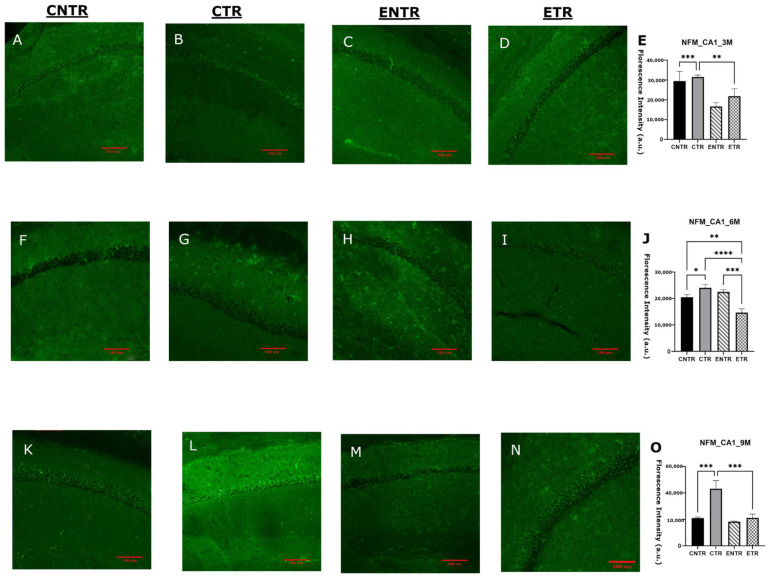
Analysis of NF-M expression in the hippocampal CA1 region at 3, 6, and 9 months of age using immunofluorescence. Representative images for CNTR, CTR, ENTR, and ETR groups are shown at 3 months (**A**–**D**), 6 months (**F**–**I**), and 9 months (**K**–**N**). Corresponding quantification of PSD-95 fluorescence intensity density is presented in (**E**), (**J**), and (**O**), respectively. Data are presented as the mean ± SEM (*n* = 3 animals per group). Statistical analysis was performed using one-way ANOVA followed by Tukey’s post hoc test. * *p* < 0.05, ** *p* < 0.01, *** *p* < 0.001, **** *p* < 0.0001. Images were acquired at 20× magnification; scale bar = 100 μm.

**Figure 10:**
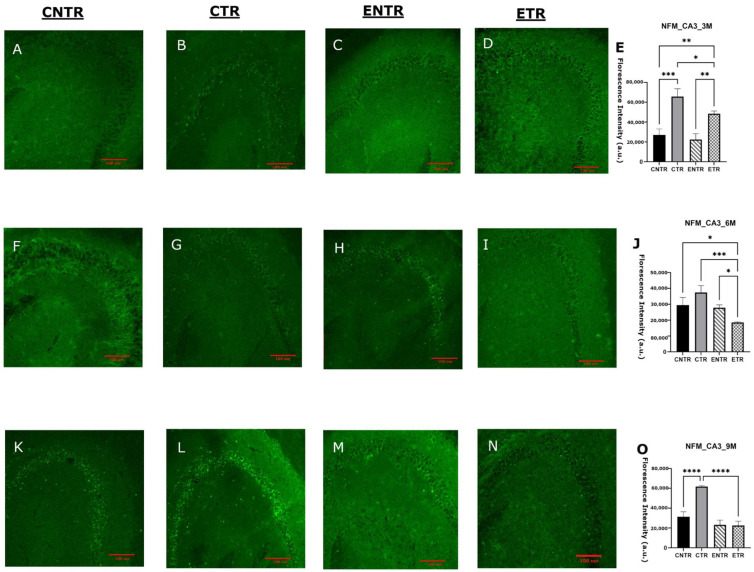
Analysis of NF-M expression in the hippocampal CA3 region at 3, 6, and 9 months of age using immunofluorescence. Representative PSD-95 immunofluorescence images are shown for 3 months (**A**–**D**), 6 months (**F**–**I**), and 9 months (**K**–**N**), with corresponding quantitative analyses presented in (**E**), (**J**), (**O**), respectively. Data are presented as the mean ± SEM (*n* = 3 animals per group). Statistical analysis was performed using one-way ANOVA followed by Tukey’s post hoc test. * *p* < 0.05, ** *p* < 0.01, *** *p* < 0.001, **** *p* < 0.0001. Images were acquired at 20× magnification; scale bar = 100 μm.

**Figure 11:**
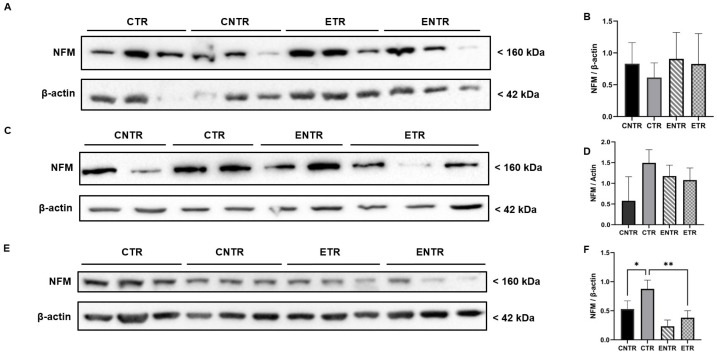
Analysis of NF-M expression in hippocampal homogenates at 3, 6, and 9 months of age using Western blot. Representative Western blot images of NF-M expression (3 months, (**A**); 6 months, (**C**); 9 months, (**E**)). Quantification of NF-M band intensities normalized to β-actin for the corresponding age groups (**B**,**D**,**F**), respectively. Data are presented as the mean ± SEM (*n* = 4 animals per group). Statistical analysis was performed using one-way ANOVA followed by Tukey’s post hoc test. * *p* < 0.05, ** *p* < 0.01.

In contrast to SYP and PSD-95 expression, NF-M expression was upregulated in the hippocampus tissues. CTR animals exhibited significantly higher immunofluorescence staining intensity of NF-M than CNTR and ETR animals in both the CA1 and CA3 hippocampal regions of all age groups (measured by immunofluorescence analysis). At 6 months of age, CNTR and ENTR animals also showed significantly elevated NF-M intensity relative to ETR animals. By 9 months of age, CTR animals continued to display significantly higher NF-M immunoreactivity than both CNTR and ETR animals (Figure 9O). In the CA3 region, by 9 months of age, CTR mice demonstrated markedly higher NF-M immunoreactivity than both CNTR and ETR mice (Figure 10O). The evaluation of NF-M expression in hippocampal homogenates also revealed age-dependent group differences by Western blot analysis. Although NF-M expression was not significant in any group at 3 months of age, group-dependent differences became more pronounced at 6 months of age. NF-M expression was higher in CTR mice compared with CNTR mice, and ENTR mice also exhibited elevated NF-M levels relative to CNTR animals. By 9 months of age, CTR animals exhibited modestly higher NF-M expression compared with CNTR animals, and significantly higher levels compared with ETR animals (Figure 11). The Western blot analysis and immunofluorescence results demonstrated that pathological NF-M accumulation increases with disease progression. Furthermore, the results suggested that early-stage dietary supplementation with neuronal membrane precursors can achieve partial attenuation in later stages.

## 3. Discussion

In this study, we investigated whether nutritional supplementation restricted to prenatal and lactational periods could exert long-term effects on behavioral performance and synaptic integrity in the 5XFAD mouse model of AD. By combining behavioral assessments with molecular analyses across multiple age groups, our study demonstrated the influence of early-life nutritional exposure on disease-relevant phenotypes later in life. Behavioral alterations emerged most clearly at later disease stages, reflecting the age-dependent progression of amyloid pathology. Motor outcomes followed a distinct trajectory: locomotion remained largely intact, whereas age-related reductions in rotarod performance aligned with previous findings of progressive motor dysfunction in 5XFAD mice [25]. Anxiety-like behavior decreased with age, in line with previous observations of reduced avoidance and behavioral disinhibition in this model [21,26,27], and showed partial sensitivity to nutritional supplementation, suggesting greater plasticity in affective circuits relative to motor pathways. In parallel, the emergence of nociceptive hypersensitivity in 5XFAD mice at 9 months of age aligns with evidence of altered pain modulation in AD patients and transgenic models [28,29], although this phenotype appeared largely resistant to supplementation. In the Morris water maze, 9-month-old 5xFAD mice displayed inefficient learning rather than overt memory impairment, with preserved probe performance, indicating a dissociation between impaired acquisition dynamics and intact memory retention, a phenomenon widely reported in amyloid-based AD models, where acquisition deficits often precede measurable impairments in reference memory [30,31]. This pattern may reflect differential involvement of hippocampal circuits underlying spatial learning and memory retention at this stage of pathology. The impairment in acquisition suggests disruption of synaptic plasticity mechanisms required for learning, whereas the preserved probe performance indicates that spatial memory traces may remain relatively intact. These findings suggest that, at this stage of disease progression, learning processes may be more vulnerable than memory retention, potentially due to partial compensatory network activity or differential susceptibility of hippocampal subfields. Together, these findings indicate that early-life nutritional intervention selectively modulates behavioral domains with retained circuit plasticity, while later-stage impairments in motor and nociceptive pathways remain comparatively refractory. It remains possible that the behavioral impact of early-life supplementation could become more efficient at later stages of disease progression, when pathological burden and circuit vulnerability are further amplified.

Consistent with this interpretation, the molecular findings further suggest that such behavioral plasticity may reflect a temporally restricted capacity for synaptic modification, as indicated by the transient stabilization of the presynaptic marker synaptophysin following early nutritional exposure. At the molecular level, presynaptic integrity was assessed by synaptophysin expression. The region- and stage-dependent reduction of synaptophysin in 5XFAD mice supports the possibility that presynaptic alterations in AD may be spatially heterogeneous and temporally constrained. The earlier and more pronounced change seen in the CA3 region compared to the CA1 region aligned with the idea that the different parts of the hippocampus are affected by amyloid-related stress in the synapses. This may be due to differences in how the circuits are built, the demands on the local cells, or the amount of amyloid in the local area. This pattern suggests that presynaptic degeneration is not uniform across the hippocampus and may preferentially compromise circuits critical for early network dysfunction. The apparent, but transient, stabilization of synaptophysin during mid-stage pathology following partial nutritional exposure indicates that presynaptic integrity may remain modifiable within a limited disease window. However, the convergence toward synaptophysin loss at later stages of disease progression across all transgenic groups suggests that once synaptic degeneration is established, it becomes increasingly resistant to nutritional modulation. This temporal profile aligns with reports that synaptic loss in 5XFAD mice closely tracks intraneuronal Aβ42 accumulation and precedes widespread synaptic protein depletion and synaptic terminal damage [32].

Importantly, the discrepancy observed between region-specific immunofluorescence findings and total hippocampal synaptophysin levels measured by Western blot likely reflects methodological differences in spatial resolution between the two approaches. Immunofluorescence allows the detection of localized synaptic alterations within specific hippocampal subfields, whereas Western blot analysis quantifies protein levels from homogenized whole-tissue samples. As a result, early or subfield-restricted synaptic pathology may be diluted in bulk hippocampal measurements, potentially masking subtle regional changes and underestimating both disease severity and the impact of nutritional intervention. This methodological limitation may also contribute to discrepancies reported across studies assessing synaptic markers using whole-tissue analyses and highlights the importance of incorporating region-specific approaches when investigating synaptic pathology in neurodegenerative models. Nutritional precursors, including DHA-based strategies, have been shown to influence synaptic protein expression in both healthy rodents and AD models [33,34]. However, their impact on presynaptic integrity within an AD context remains poorly defined, particularly during the prenatal and lactation period. The present findings suggest that combined UMP, choline, and fish oil supplementation may exert modest, region-specific effects on synaptophysin levels but that these effects are limited in duration and insufficient to counteract progressive presynaptic degeneration at advanced stages of pathology. The progressive reduction of PSD-95 in 5XFAD mice supports the view that postsynaptic destabilization is a core component of Alzheimer’s disease-related synaptic pathology [23]. The observed decline across disease stages, therefore, reflects increasing postsynaptic vulnerability rather than simple neuronal loss. The relative preservation of PSD-95 in supplemented animals, particularly at the earlier stages of disease, suggests that postsynaptic compartments may respond more robustly than presynaptic terminals to nutritional support, although this protective capacity appears to diminish as pathology progresses. Supplementation was associated with an early relative preservation of PSD-95; this effect was not sustained during subsequent disease progression, but higher PSD-95 levels were again observed in supplemented animals at late-stage ages. In 5XFAD mice, PSD-95 loss in apical dendrites emerges despite preserved neuronal numbers, reflecting postsynaptic degeneration rather than cell loss [35], and is closely linked to amyloid exposure and impaired excitatory synaptic transmission [36,37]. Progressive PSD-95 downregulation has been reported in aged 5XFAD mice and across cortical regions in humans, where it correlates with cognitive impairment [38,39,40,41]. Nutritional precursors such as DHA, UMP, and choline enhance membrane phospholipid synthesis and upregulate synaptic markers, including PSD-95 [33,42,43]. Although these interventions do not normalize synaptic architecture, their ability to attenuate PSD-95 loss suggests a limited capacity to mitigate postsynaptic alterations.

The NF-M, a core component of the axonal cytoskeleton, reflects axonal integrity and conduction capacity and is dysregulated in neurodegenerative conditions, including AD [24,44]. Consistent with reports linking elevated neurofilament concentrations to axonal injury [44], NF-M levels increased in a genotype-dependent manner in 5XFAD mice, reflecting a compensatory mechanism and neurofilament accumulation. This elevation was evident early in our study and became more pronounced with disease progression. Notably, our findings suggest a region-specific increase in NF-M within the CA3 hippocampal subfield, which may indicate increased axonal susceptibility in this region. Across hippocampal regions, supplemented animals exhibited lower NF-M levels compared with transgenic mice, suggesting partial attenuation of pathological neurofilament accumulation, although disease progression was not prevented.

At the whole-hippocampus level, early subfield-specific alterations in NF-M were not detected; however, pronounced elevations became evident as pathology progressed, underscoring that global tissue measurements can regionally mask early axonal degeneration. As indicated by the extant literature, elevated neurofilament levels have been widely associated with axonal injury [44]. Our findings confirmed substantial axonal pathology in transgenic mice and indicated that supplementation reduces (but does not fully prevent) pathological NF-M accumulation in middle- and late-aged animals. This attenuation suggests a potential stabilizing effect on axonal structures, possibly by limiting the upstream processes that drive NF-M hyperphosphorylation, a key contributor to axonal degradation in AD. Although the direct influence of UMP, choline, or fish oil on NF-M phosphorylation has not yet been investigated, their well-established roles in enhancing phospholipid synthesis [45], improving axonal membrane stability [46], and reducing oxidative stress [47] offer a plausible mechanistic framework for the observed reduction in NF-M accumulation. This interpretation is further supported by evidence that choline, UMP, and DHA can mitigate oxidative damage and preserve axonal integrity [19,24].

Some limitations of our study should be acknowledged. Including additional brain regions and extended age groups would provide more detailed information regarding synaptic alterations in neurodegenerative processes. Due to the limited availability of animals, we were unable to perform sex-based analyses. Inclusion of sex as a biological variable would have required substantially larger group sizes across multiple treatment and age cohorts, which was beyond the scope of the present study. Consequently, potential sex-dependent differences in the response to early-life supplementation cannot be ruled out and should be systematically addressed in future studies designed to evaluate sex-specific trajectories of synaptic and behavioral alterations in AD models. In addition, our control animals were transgenic littermates rather than wild types. Although our results revealed alterations in PSD-95, synaptophysin, and NF-M expression between non-transgenic and transgenic animals, comparing transgenic mice with wild-type controls would have increased the consistency of our findings and minimized genetic background variability. Despite these limitations, we believe that our findings still offer valuable insights into synaptic alterations associated with neurodegeneration.

To our knowledge, this study is the first to investigate the long-term effects of choline, UMP, and fish oil supplementation during the prenatal and lactation periods in 5XFAD mice using an integrated molecular and multidimensional behavioral framework across multiple disease stages, specifically at 3, 6, and 9 months of age. Since molecular and behavioral measures were obtained from different animals, the findings should be interpreted as associative rather than directly linked. Although supplementation during early life did not halt the overall progression of the disease, it consistently attenuated synaptic and axonal pathology in parallel with selective changes in behavioral performance, ensuring partial preservation of key markers of synaptic integrity, including synaptophysin, PSD-95, and NF-M, and yielding molecular profiles similar to those of healthy controls. Importantly, this study provides a longitudinal evaluation of early-life nutritional intervention, demonstrating sustained molecular and functional effects across distinct stages of AD-like pathology, and combines behavioral and region-specific molecular analyses to characterize the temporal progression of synaptic and axonal alterations in the 5xFAD model.

## 4. Materials and Methods

### 4.1. Ethical Approval

All procedures involving animals were conducted in accordance with the National Institutes of Health Guide for the Care and Use of Laboratory Animals and complied with the ARRIVE guidelines. All experimental protocols were approved by the Acıbadem Mehmet Ali Aydınlar University Animal Experiments Local Ethics Committee (ACU-HADYEK, approval no. 2019/46).

### 4.2. Animal Model

The 5XFAD transgenic mouse line [38], which harbors five familial AD-related mutations (APP: Sweden, Florida, and London; PSEN1: M146L and L286V) on a mixed C57BL/6 × SJL genetic background (strain designation: B6SJL-Tg), was obtained from the Jackson Laboratory (Jackson Laboratory, Bar Harbor, ME, USA) (MMRRC_034840-JAX; stock no. 034848-JAX).

### 4.3. Diet Regimen, Experimental Design, and Breeding

For the experimental diet (Research Diets #19022605), we used a modified AIN-76 formula containing 19.9% protein, 64.8% carbohydrate, and 5.8% fat. Specifically, this diet consisted of casein (200 g/kg), DL-methionine (3 g/kg), corn starch (150 g/kg), sucrose (500 g/kg), cellulose (50 g/kg), corn oil (48.99 g/kg), mineral mix S10001 (35 g/kg), vitamin mix V10001 (10 g/kg), fish oil (0.3%) [48,49], uridine monophosphate (0.5%) [48], and choline chloride (5000 mg/kg) [50,51]. The control diet (Research Diets #D19022603) was based on a standard AIN-76 formulation [52] and contained 1100 mg/kg of choline chloride, reflecting the recommended nutritional level [50,51]. The diets were stored at −20 °C in vacuum-sealed bags and provided fresh daily to prevent oxidation.

At 3 months of age, female 5XFAD mice were mated with non-transgenic C57BL/6J males and randomly assigned to either the experimental or control diet. Females consumed their assigned diet before mating, throughout gestation, and until weaning. Offspring remained with their dams until postnatal day 21 (P21) [51], after which they were genotyped and classified as transgenic (TR) or non-transgenic (NTR). These offspring were then assigned to either the experimental or control diet, yielding four groups: ETR (experimental diet, transgenic), ENTR (experimental diet, non-transgenic), CTR (control diet, transgenic), and CNTR (control diet, non-transgenic). All animals were maintained on their control diets until they were euthanized.

Control animals consisted of transgenic littermates rather than wild-type animals in order to minimize variability related to genetic background and environmental factors. This design allowed evaluation of the effects of early-life supplementation within the transgenic model while maintaining comparable housing and developmental conditions across experimental groups.

### 4.4. Genotyping

Animals were obtained by interbreeding C57BL/6 and SJL mouse lines. For genomic DNA isolation, ear tissue samples were collected and subsequently used for genotype determination via PCR. Primer information is provided in Supplementary File S1. Amplification was initiated with denaturation at 94 °C for 3 min, followed by 35 cycles comprising denaturation at 94 °C for 30 s, annealing at 57.3 °C for 60 s, and elongation at 72 °C for 60 s, followed by a final extension step at 72 °C for 2 min. PCR amplicons were analyzed by electrophoresis on 1.5% agarose gels prepared in Tris-acetate-EDTA buffer, supplemented with 1× loading dye (NEB B7021-S), and separated at 100 V for 45 min before visualization (Supplementary File S1).

### 4.5. Animal Housing and Husbandry

All animals were housed in the Laboratory Animal Application and Research Center of Acıbadem Mehmet Ali Aydınlar University (DEHAM, Istanbul, Turkey). Mice were maintained under controlled conditions with a 12 h light/dark cycle and an ambient temperature of 22–25 °C in a temperature-controlled room with ambient humidity and ad libitum access to food and water. Animals were group-housed (4–5 per cage) in individually ventilated cages to minimize environmental stress and ensure standardized husbandry conditions.

### 4.6. Disease Progression and Euthanasia

**Figure 12:**
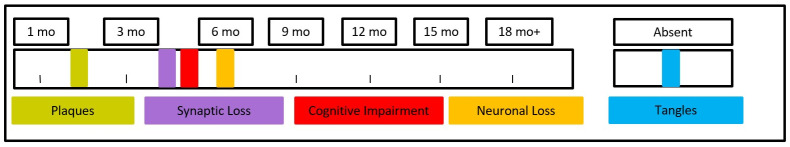
Phenotypic characterization of 5XFAD (BS6JL-Tg) mice [53].

To assess the temporal progression of Alzheimer’s disease-related pathology, mice were euthanized at 3, 6, and 9 months of age, corresponding to early, mid-, and late-stage phenotypic manifestation in the 5XFAD model (Figure 12). These points were selected based on established reports characterizing age-dependent pathology in 5XFAD mice [53].

### 4.7. Cognitive and Behavioral Assessment

Mice in the four groups, CNTR (*n* = 7), CTR (*n* = 5), ENTR (*n* = 6), and ETR (*n* = 7), were subjected to behavioral testing. A comprehensive behavioral test battery was administered to evaluate cognitive and behavioral functions in all groups of 5XFAD mice. The battery included the open field test (locomotor activity), the elevated plus maze (anxiety-like behavior), the rotarod test (motor coordination and balance), the Morris water maze (spatial learning and memory), and the paw withdrawal test (thermal nociception). To reduce stress- and experience-dependent interference between paradigms, a minimum interval of 48 h was maintained between successive behavioral tests.

#### 4.7.1. Open Field Test

Locomotor activity was assessed using a 50 × 50 cm open-field arena. Mice were placed individually in the center or in one corner of the arena and allowed to explore freely for 5 min. The field was divided into 16 equal quadrants, and locomotion was quantified as the total number of squares visited [54].

#### 4.7.2. Elevated Plus Maze Test

Anxiety-like behavior was assessed using a standard elevated plus maze. The apparatus consisted of two open arms (50 × 10 cm) and two closed arms (50 × 10 × 40 cm) extending from a central platform (10 × 10 cm), elevated 50 cm above the floor. Each mouse was placed in the central zone and allowed to explore for 5 min. Time spent in the open and closed arms was recorded to assess anxiety [55].

#### 4.7.3. Rotarod Test

The rotarod test is a valuable tool for assessing coordination, balance, and motor learning in animal models of neurodegenerative disease, such as AD [56]. In the present study, motor coordination and balance were assessed using an accelerating rotarod apparatus [57,58] to evaluate motor function across different age groups and provide insights into potential age-related or disease-specific impairments.

Mice were acclimated to the apparatus by being placed on a fixed rod, with resistance set to the maximum level for one session before testing. Mice were trained to acclimate to the apparatus on the day before testing by running on the rod, which accelerated from 4 rpm to 10 rpm over a duration of 5 min. On the test day, mice underwent three trials, with an inter-trial interval of 30 min to prevent fatigue. During each trial, mice were placed onto the rotating rod facing forward, and the rod accelerated linearly from 4 rpm to 40 rpm over a total duration of 5 min. Motor performance was evaluated by measuring the latency to fall, defined as the time the animal remained on the rotating rod before falling. The fall of each mouse automatically triggered the trip plate, stopping the timer and recording the latency. For each mouse, the mean latency across the three trials was calculated and used for further analysis. Motor coordination was expressed as the falling latency, with longer latencies indicating better motor performance.

#### 4.7.4. Morris Water Maze Test

Spatial learning and memory were assessed using a five-day Morris water maze protocol. The apparatus consisted of a circular tank (180 cm in diameter, 60 cm in height) filled with water, maintained at 24–26 °C. A circular escape platform was positioned in a fixed location and submerged 1 cm below the surface. During the acquisition phase (days 1–4), mice underwent two training sessions per day. For each trial, mice were placed in the water at four designated points and allowed up to 90 s to locate the platform. Animals that failed to reach the platform within this time were allowed to remain on the platform for 30 s to facilitate the encoding of spatial cues placed around the maze.

Escape latency from each trial was recorded, and daily means were calculated for analysis. On day five, a probe trial was conducted to evaluate memory retention: the platform was removed, and mice were allowed to swim freely for 90 s. Time spent in the target quadrant and the number of crossings over the former platform location were quantified as indices of spatial memory [59,60,61].

#### 4.7.5. Paw Withdrawal Test

Thermal nociception was assessed using a radiant heat paw withdrawal assay. Mice were habituated to the testing apparatus for 30 min prior to testing to minimize stress-induced variability. A focused radiant heat stimulus was applied to the plantar surface of each hind paw, and withdrawal latency was recorded as a measure of heat sensitivity. The procedure was repeated three times at 5-min intervals, and the average withdrawal latency was calculated. To avoid tissue damage, the cut-off time was set to 20 s [62].

### 4.8. Perfusion and Tissue Preparation

Mice were deeply anesthetized with isoflurane (1–2% in oxygen), and an adequate depth of anesthesia was confirmed by the loss of movement and tail pinch reflex prior to transcardial perfusion. Animals were transcardially perfused with 10–15 mL of heparinized phosphate-buffered saline (PBS), followed by 25 mL of 4% paraformaldehyde (PFA) in 0.01 M PBS (pH 7.4) delivered at approximately 5 mL/min. Brains were removed and cryoprotected in graded sucrose solutions (10%, 20%, 30% *w*/*v*) at 4 °C until they sank. Each hemisphere was embedded in Tissue-Tek OCT (Sakura^®^ Finetek, Torrance, CA, USA), rapidly frozen, and stored at −20 °C for immediate sectioning or at −80 °C for long-term preservation [63]. Animals were euthanized following transcardial perfusion in accordance with institutional and national guidelines.

### 4.9. Immunofluorescence: Experimental Scope

Sagittal brain sections collected at 3, 6, and 9 months were processed from all four experimental groups (ETR, ENTR, CTR, CNTR) to assess SYP, PSD-95, and NF-M immunoreactivity. This design enabled comparison of presynaptic (SYP), postsynaptic (PSD-95), and axonal integrity (NF-M) across disease progression.

### 4.10. Tissue Processing and Immunofluorescence Labeling

Immunofluorescence analysis was performed on three animals per group (*n* = 3). Sagittal brain sections containing the hippocampal cornu ammonis (CA1–CA3) subfields were collected using coordinates guided by the Allen Mouse Brain Atlas [64]. Sagittal brain sections were cut at 30 µm using a cryostat. Free-floating sections were post-fixed in 4% PFA in PBS for 15 min and rinsed three times in PBS (5 min each). Sections were then blocked in 5% bovine serum albumin (BSA) in PBS for 60 min at room temperature and incubated overnight at 4 °C with primary antibodies diluted in PBS containing 1% BSA and 0.3% Triton X-100: synaptophysin (SYP, 1:100, CST #36406), PSD-95 (1:900, CST #3450S), and neurofilament medium chain (NF-M, 1:100, BosterBio #M06821-1). After primary incubation, sections were washed three times in PBS and incubated for 1 h at room temperature in the dark with Alexa Fluor-conjugated secondary antibodies (anti-rabbit Alexa 488, 1:500; anti-mouse Alexa 568, 1:1000; Thermo Fisher Scientific, Waltham, MA, USA). After the final PBS washes, sections were mounted onto poly-L-lysine-coated glass slides using Fluoroshield mounting medium containing DAPI (Abcam, ab104139) (Table 1).

### 4.11. Immunofluorescence Imaging and Quantification

Immunofluorescence images were acquired using a Zeiss LSM 700 confocal microscope (Carl Zeiss, Thornwood, NY, USA) with a 20× objective. Fluorophores were excited using 488 nm and 555 nm laser lines. The pinhole diameter was set to 1 Airy unit for all image acquisitions. Images were collected from the CA1 and CA3 hippocampal subfields to evaluate the distribution and expression of SYP, PSD-95, and NF-M. All images were acquired using identical microscope settings across experimental groups. Fluorescence intensity within defined regions of interest (ROIs) using ImageJ 1.54 P (NIH, Bethesda, MD, USA). Fluorescence images were acquired under identical imaging conditions for all groups. Values were expressed as relative optical density.

### 4.12. Detection of Amyloid-β Deposits Using Congo Red Staining

To visualize amyloid-β (Aβ) plaques, Congo red staining was conducted. Frozen brain sections were mounted onto lysine-coated slides, air-dried for approximately 5 min, and post-fixed in 4% PFA for 15 min at room temperature. The staining procedure followed that described in a previous study [65], with minor modifications. After staining with Congo Red (Sigma Aldrich, St. Louis, MO, USA), sections were rinsed, dehydrated, and cover-slipped using Entellan (Merck, Darmstadt, HE, Germany). The presence of Aβ plaques in the Congo red-stained hippocampal area was assessed manually under a Zeiss LSM 700 confocal microscope (Zeiss, Oberkochen, Germany) (Supplementary File S2). The Aβ plaque burden in the hippocampal region was evaluated manually under a Zeiss LSM 700 microscope (Oberkochen, Germany) [65] (Supplementary File S2).

### 4.13. Western Blotting

Hippocampal tissues were rapidly dissected from isoflurane-anesthetized mice and snap-frozen in liquid nitrogen before storage at −80 °C. Western blot analyses were performed on samples from four animals per group. Tissues were homogenized in ice-cold RIPA buffer (50 mM Tris-HCl, pH 7.4, 150 mM NaCl, 1% NP-40, 0.25% sodium deoxycholate, 1 mM EDTA) supplemented with phenylmethylsulfonyl fluoride (PMSF), sodium orthovanadate, sodium fluoride, and a protease inhibitor cocktail. Homogenates were processed using a Dounce homogenizer, briefly sonicated in an Omni Ruptor 4000 sonicator for approximately 1 s, and centrifuged at 14,000× *g* for 15 min at 4 °C for collection of the supernatant. Protein concentrations were determined using the Bradford assay (Bio-Rad, Hercules, CA, USA). In brief, equal quantities of protein (30 μg) were separated on 10–12% SDS-PAGE gels and transferred to nitrocellulose or PVDF membranes using a semi-dry transfer system (Bio-Rad Trans-Blot; 25 V, 40 min). Membranes were blocked in 5% BSA for 1 h at room temperature and incubated overnight at 4 °C with primary antibodies against NF-M (1:1000, BosterBio, Pleasanton, CA, USA), synaptophysin (SYP, 1:1000, CST, Danvers, MA, USA), PSD-95 (1:1000, Thermo Fisher, Waltham, MA, USA), and β-actin (1:5000, Thermo Fisher, Waltham, MA, USA). After washing in Tris-buffered saline with Tween-20 (TBS-T), membranes were incubated for 1 h at room temperature with HRP-conjugated secondary antibodies (1:5000; anti-rabbit for PSD-95 and SYP, anti-mouse for NF-M and β-actin) (Table 1). Protein bands were visualized using enhanced chemiluminescence (SuperSignal™ West Femto Maximum Sensitivity Substrate, Thermo Fisher Scientific, USA) and quantified in Image Lab (6.0.1 Bio-Rad, Hercules, CA, USA) and ImageJ 1.54s (RRID: SCR_003070, NIH, USA) software.

### 4.14. Statistical Analysis

All statistical analyses were performed in GraphPad Prism version 10.2.0. Data were first assessed for normality using the Shapiro–Wilk test to determine the appropriateness of parametric versus non-parametric statistical approaches. Details of the specific statistical tests, post hoc comparisons, and significance thresholds used for each dataset are reported in the corresponding figure legends. The data were calculated as the mean ± SEM (unless otherwise indicated in the figure), and statistical significance was set at *p* < 0.05. Between-group differences were analyzed using one-way ANOVA for normally distributed data or Kruskal–Wallis tests for non-parametric data. Tukey’s post hoc test (ANOVA) or Dunn’s test (Kruskal–Wallis) was applied where appropriate. Escape latencies across training days were analyzed using a two-way repeated-measures ANOVA (group × day), followed by Bonferroni post hoc tests for multiple comparisons. Probe trial performance was analyzed using one-way ANOVA with Tukey’s post hoc test.

## 5. Conclusions

The multidimensional approach used in this study provides a comprehensive characterization of how early-life nutritional support can modulate various functional domains affected during neurodegeneration. These results are consistent with previous studies that showed that phospholipid precursors increase synaptic protein expression and plasticity; the current findings extend this evidence by demonstrating that nutritional exposure to early developmental periods can create lasting molecular and functional effects in later life. Overall, our data support the concept that early-life nutritional programming may modulate long-term synaptic integrity and functional resilience in an AD model.

## Figures and Tables

**Table 1:** Antibody list.

Antibodies	Source	Catalog Number/Dilution
NF-M	BosterBio	#M06821-1/IF 1:100/WB 1:1000
SYP	CST	#36406/IF 1:100/WB 1:1000
PSD-95	CST	#3450S/IF 6:900/WB 1:1000
β-Actin Loading Control Monoclonal Antibody (BA3R)	Thermo Fisher Scientific	MA5-15739/WB 1:5000
Peroxidase-conjugated Anti-Rabbit IgG	Jackson ImmunoResearch	#111-035-003/WB 1:5000
Peroxidase-conjugated Anti-Mouse IgG	Jackson ImmunoResearch	#115-035-003/WB 1:5000

BosterBio (BosterBio, Pleasanton, CA, USA); CST (Cell Signaling Technology (CST), Danvers, MA, USA); Thermo Fisher Scientific (Thermo Fisher Scientific, Waltham, MA, USA); Jackson ImmunoResearch (Jackson ImmunoResearch Laboratories, West Grove, PA, USA).

## Data Availability

The original contributions presented in this study are included in the article/Supplementary Material. Further inquiries can be directed to the corresponding author.

## Supplementary Materials

The following supporting information can be downloaded at: https://www.mdpi.com/article/10.3390/ijms27083342/s1.

## Abbreviations

The following abbreviations are used in this manuscript:

PSD-95	Postsynaptic density protein 95
SYP	Synaptophysin
NF-M	Neurofilament medium chain
PCR	Polymerase chain reaction
DHA	Docosahexaenoic acid
UMP	Uridine monophosphate
NTR	Non-transgenic
TR	Transgenic
CNTR	Control diet, non-transgenic
CTR	Control diet, transgenic animal
ENTR	Experimental diet, non-transgenic
ETR	Experimental diet, transgenic
AD	Alzheimer’s Disease
Aβ	Amyloid-β
MWM	Morris water maze
PFA	Paraformaldehyde
PBS	Phosphate-buffered saline
BSA	Bovine serum albumin
