# Supplementary material for: Effects of UMP, Choline, and Fish Oil on Synaptic Integrity and Motor Coordination in an Alzheimer’s Disease Mouse Model
Source: Int J Mol Sci. 2026 Apr 8;27(8):3342. doi: 10.3390/ijms27083342 (PMC13116225; doi:10.3390/ijms27083342)
Supplement: Supplementary file 1 [file ijms-27-03342-s001.zip › Supplementary File_IJMS/Supplementary material File S1.pdf]

Sup. Table S1: Primers used in 5xFAD genotyping

Primer Name	Base Sequence (5’ -> 3’)	Base Lenght	Tm (°C)	Product Length
Control_Forward	CTA GGC CAC AGA ATT GAA AGA TCT	24	59,3	324
Control_Reverse	GTA GGT GGA AAT TCT AGC ATC ATC C	25	61,3	324
APP_Forward	AGG ACT GAC CAC TCG ACC AG	20	61,4	377
APP_Reverse	CGG GGG TCT AGT TCT GCA T	19	58,8	377
PSEN1_Forward	AAT AGA GAA CGG CAG GAG CA	20	57,3	608
PSEN1_Reverse	GCC ATG AGG GCA CTA ATC AT	20	57,3	608

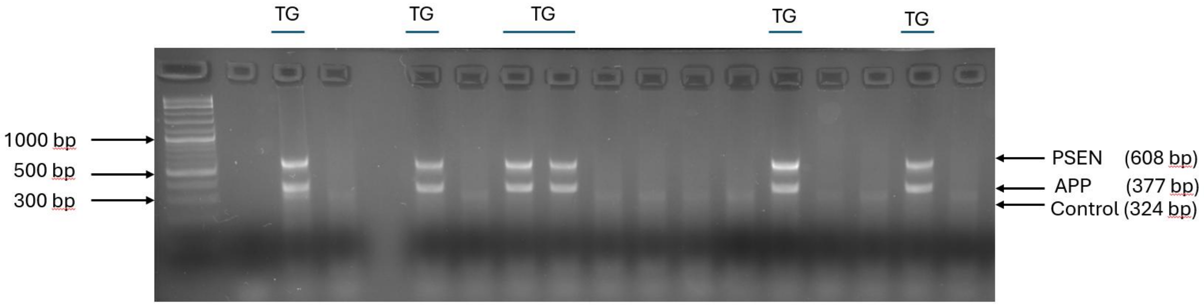

Sup. Fig. S1: Figure that show the PCR products' results from agarose gel electrophoresis
