# Supplementary material for: Effects of UMP, Choline, and Fish Oil on Synaptic Integrity and Motor Coordination in an Alzheimer’s Disease Mouse Model
Source: Int J Mol Sci. 2026 Apr 8;27(8):3342. doi: 10.3390/ijms27083342 (PMC13116225; doi:10.3390/ijms27083342)
Supplement: Supplementary file 1 [file ijms-27-03342-s001.zip › Supplementary File_IJMS/Supplementary material File S2.pdf]

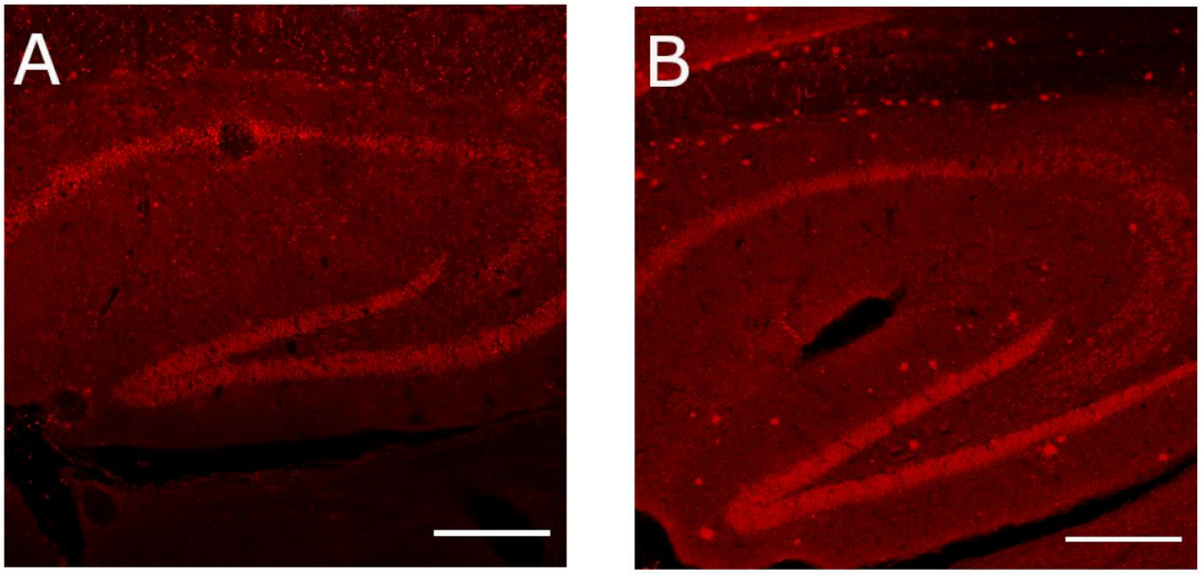

Sup. Fig. S2. Amyloid Beta Accumulation in 5xFAD Alzheimer's Transgenic Mouse Model

Representative images demonstrate amyloid-β plaque deposition in the hippocampus of the 5xFAD Alzheimer's disease mouse model. (A) Congo red–stained sections from transgenic-negative mice show no detectable amyloid-β deposition. (B) Congo red–stained hippocampal sections from transgenic-positive mice show marked amyloid-β accumulation with prominent dense-core plaques. Scale bar: 200 μm.
